# Effect of temperature and nutrients on the growth and development of seedlings of an invasive plant

**DOI:** 10.1093/aobpla/plv044

**Published:** 2015-04-29

**Authors:** Hana Skálová, Lenka Moravcová, Anthony F. G. Dixon, P. Kindlmann, Petr Pyšek

**Affiliations:** 1 Department of Invasion Ecology, Institute of Botany, The Czech Academy of Sciences, CZ-252 43, Průhonice, Czech Republic; 2 School of Biological Sciences, University of East Anglia, Norwich NR4 7TJ, Norfolk, UK; 3 Department of Biodiversity Research, Global Change Research Centre, The Czech Academy of Sciences, Bělidla 4a, CZ-602 00, Brno, Czech Republic; 4 Institute for Environmental Studies, Charles University in Prague, Benátská 2, CZ-128 01, Prague, Czech Republic; 5 Department of Ecology, Faculty of Science, Charles University in Prague, Viničná 7, CZ-128 44, Prague, Czech Republic

**Keywords:** *Ambrosia artemisiifolia*, common ragweed, invasive species, non-indigenous plants, nutrient limitation, plant nutrition, rate of development, thermal time

## Abstract

Plant species distributions are determined by the response of populations to regional climates; however, little is known about how alien plants that arrive in central Europe from climatically warmer regions cope with the temperature conditions at the early stage of population development. *Ambrosia artemisiifolia* (common ragweed) is an invasive annual plant causing considerable health and economic problems in Europe. Although climate-based models predict that the whole of the Czech Republic is climatically suitable for this species, it is confined to the warmest regions. To determine the factors possibly responsible for its restricted occurrence, we investigated the effects of temperature and nutrient availability on its seedlings. The plants were cultivated at one of seven temperature regimes ranging from 10 to 34 °C, combined with three nutrient levels. The data on the rate of leaf development were used to calculate the lower developmental threshold (LDT, the temperature, in °C, below which development ceases), the sum of effective temperatures (SET, the amount of heat needed to complete a developmental stage measured in degree days above LDT) and width of the thermal window. The rate of development decreased with decrease in temperature and nutrient supply. Besides this, the decrease in the availability of nutrients resulted in decreased LDT, increased SET and wider thermal window. The dependence of LDT and SET on the availability of nutrients contradicts the concept that thermal constants do not vary. Our results highlight temperature as the main determinant of common ragweed's distribution and identify nutrient availability as a factor that results in the realized niche being smaller than the fundamental niche; both of these need to be taken into account when predicting the future spread of *A. artemisiifolia*.

## Abstract (precis)

Little is known about how alien plants that arrive in central Europe from climatically warmer regions cope with the temperature conditions at the early stage of population development. Using *Ambrosia artemisiifolia* (common ragweed), an invasive annual plant as a model, we found that the rate of seedling development decreased with decrease in temperature and nutrient supply. Our results thus highlight temperature as the main determinant of common ragweed's distribution and identify nutrient availability as a factor that results in the realized niche being smaller than the fundamental niche.

## Introduction

Plant species distributions are determined by the response of populations to regional climates. Alien plants, many of which arrive in regions from areas with a warmer climate, provide a suitable model for studying the effect of temperature on determining their distribution. Since the early stage of population development is crucial for determining whether or not a species can successfully establish in a given region (Richardson *et al*. 2000; Blackburn *et al*. 2011), knowing the response of seedlings to temperature can greatly improve our understanding of invasion and provide knowledge necessary for the efficient management of invasive species.

Common ragweed (*Ambrosia artemisiifolia*) is an invasive wind-pollinated annual plant causing considerable health and economic problems (Rybníček and Jäger 2001; Chauvel *et al*. 2006; Kömives *et al*. 2006; Fumanal *et al*. 2007; Essl *et al*. 2009; Šikoparija *et al*. 2009). It was introduced in Europe in the 19th century from its native range in North America, where it occurs in eastern and central USA (Kartesz and Meacham 1999; Brandes and Nitzsche 2007), and at the beginning of 2000s it was reported as a neophyte by 36 countries, in the majority of which it was naturalized (Lambdon *et al*. 2008). After an extended lag phase, it has spread since the 1940s via transportation networks and contaminated crop seed, and recently rapidly invaded central Europe (Chauvel *et al*. 2006; Brandes and Nitzsche 2007; Dullinger *et al*. 2009; Essl *et al*. 2009; Richter *et al*. 2013*a*, *b*; Martin *et al*. 2014). It is predicted that it will spread further in Europe as the species is assumed to be favoured by ongoing global warming (Richter *et al*. 2013*b*; Storkey *et al*. 2014). In its global invaded range, which includes all continents and some islands (New Zealand, Hawaii, Madagascar, Mauritius; Brandes and Nitzsche 2007), it occurs in a wide range of open and nutrient-rich, disturbed ruderal habitats and arable land (Chauvel *et al*. 2006; Essl *et al*. 2009; Pinke *et al*. 2011; Pyšek *et al*. 2012*a*).

Climate-dependent phenological models suggest that the distribution of *A. artemisiifolia* in Europe is limited by low temperatures in the North where plants are prevented from completing reproduction by autumn frosts, which kill adult plants (Chapman *et al*. 2014), and drought in the South, which inhibits seed germination and seedling emergence (Storkey *et al*. 2014). On the other hand, other environmental variables such as habitat type, land use, crop type and soil nutrients also play a role in the occurrence and abundance of *A. artemisiifolia* at a regional scale (Essl *et al*. 2009; Pinke *et al*. 2011, 2013). At a local scale, soil disturbance and removal of vegetation enhance seedling recruitment of ragweed from the soil-seed bank (Fumanal *et al*. 2008), by favouring the growth of juveniles as this species is not a good competitor (Bazzaz 1974; Leskovšek *et al*. 2012*a*). Its success as an invasive species is associated with its high production of seed (1200–2500 seeds per plant; Fumanal *et al*. 2007; Moravcová *et al*. 2010), which form a long-term persistent soil-seed bank, with seeds remaining viable for up to 40 years (Baskin and Baskin 1980).

*Ambrosia artemisiifolia* was introduced with clover seed from North America and first recorded in the Czech Republic in 1883, and is classified as an invasive neophyte (term used for species introduced after 1500 A.D.) in that country (Pyšek *et al*. 2012*b*). Naturalized populations occur only in the warmest parts of the country, i.e. in the Elbe region, and southern and northern Moravia, with casual populations scattered throughout the rest of the country (Pyšek *et al*. 2012*a*). This species prefers open dry habitats on sandy or gravel substrata with low vegetation cover. Most records are from around railway stations, river harbours, transit sheds, agricultural and industrial areas dealing now or in the past with soya beans, and neighbouring ruderal areas (Jehlík 1998).

The present distribution of *A. artemisiifolia* in the Czech Republic does not correspond to the climate-based prediction that the whole country is suitable for this species to complete its life cycle and set seed (Cunze *et al*. 2013; Chapman *et al*. 2014; Storkey *et al*. 2014). Some predictions highlight the importance of taking local environmental conditions into account (Stratonovitch *et al*. 2012). These authors indicate that this plant's response to climate change is confounded by variation in soil properties, which accords with the reported effects of soil nutrients on *A. artemisiifolia* distribution (Pinke *et al*. 2011, 2013). The role of nutrient availability is indicated by analyses of the factors that affect the distribution of this species in Europe (Pinke *et al*. 2011, 2013) and by greenhouse experiments (Leskovšek *et al*. 2012*a*, *b*), but its interaction with temperature has not been experimentally tested.

To obtain a detailed insight into the ecological factors that are likely to co-determine the performance of *A. artemisiifolia* in the field, we investigated the effect of temperature and nutrient availability on the rate of development (RD) of its seedlings. The good survival of seedlings is crucial for the establishment and successful population regeneration of annual species and there are several studies directly linking seedling traits with invasion success (Grotkopp and Rejmánek 2007; Morrison and Mauck 2007; Zheng *et al*. 2009; Skálová *et al*. 2012). The effect of temperature on seedling development has been previously studied (Granier *et al*. 2002; Gramig and Stoltenberg 2007), with one of the few studies on *A. artemisiifolia* (Deen *et al*. 1998*a*). This approach, which is based on measuring the rate of plant development at different temperatures, provides results that can be used to calculate the following characteristics: the lower developmental threshold—LDT (the temperature below which development ceases); the sum of effective temperatures—SET [the amount of heat needed to complete a developmental stage measured in degree days (DD) above the LDT] and thermal window (the difference between the minimum and maximum temperatures of the range over which development occurs) (Jarošík *et al*. 2002, 2004).

In this paper, we ask the following questions: What are the effects of temperature and nutrient availability on the RD of *A. artemisiifolia* seedlings? What is the SET and LDT for development and are they modified by nutrient availability? How does nutrient availability affect variations in the thermal window of this species? What are the effects of temperature and nutrient availability on size and biomass allocation in seedlings?

## Methods

### Experimental design

The seeds of *A. artemisiifolia* were collected at a ruderal site (48°43′35.0″N; 16°58′42.7″E) 1.4 km south-east of the town of Lanžhot in S Moravia, the Czech Republic, in October 2009. We collected ∼1000 seeds from at least 50 plants in a population of hundreds of individuals growing in an area of ∼300 m^2^ at a soil dump. After collection, the seeds were stored in paper bags at room temperature. Before the experiment, the seeds were cold-stratified in wet sand in the dark at 4 °C for 3 months and then germinated at a diurnally fluctuating temperature of 25/10 °C (day/night cycle 12 h/12 h with a corresponding light/dark alternation). Germinated seeds with a radicle length of 5–10 mm were moved to containers filled with pure silica sand. Sixteen germinated seeds were planted and grown at each of the temperatures and nutrient regimes, giving a total of 336 plants (i.e. 7 temperature regimes × 3 nutrient regimes × 16 replicates). Some of the seeds did not germinate or the seedlings died during cultivation and in total 48 individuals (14.2 % of the initial set) were lost.

The seedlings were grown in growth chambers (Vötsch 1014) under identical irradiation [14/10 h light/dark regime; photosynthetically active radiation of 360 μmol m^−2^ s^−1^, red radiation (R, *λ* = 660 nm) of 26 μmol m^−2^ s^−1^ and far-red radiation (FR, *λ* = 730 nm) of 15 μmol m^−2^ s^−1^, R/FR = 1.73; radiation measured using an SPh 2020 photometer from Optické dílny Turnov, Czech Republic] and air moisture (80 %) conditions. The seedlings were cultivated at constant temperatures of 10, 14, 18, 22, 26, 30 and 34 °C, which is the full range of temperatures possible using the above growth chambers. The three different nutrient levels used in our experiments were obtained using 10, 50 and 100 % Knop nutrient solution; 100 % Knop solution contained 152 mg N/L. To achieve a stable nutrient supply, conductivity of the solutions was measured daily and nutrient solution or demineralized water was added to keep the conductivity at 370, 1770, 3450 μS cm^−1^, respectively. In addition, the nutrient solutions were changed every second day to prevent the growth of algae.

### Recording the rate of seedling development

To record the time between the appearance of the first and seventh pair of stem leaves (excluding cotyledons), the plants were checked and measured daily (following Gramig and Stoltenberg 2007). The leaves were assumed to have appeared when their size was equal to or exceeded 1 mm. After appearance of the seventh pair of leaves, height was measured and the plants harvested. The biomass was divided into shoots and roots, dried at 70 °C for 8 h and weighed.

### Data analysis

The rate of development was defined as 1/(time in days between appearance of the first and seventh pair of stem leaves) = 1/d. For each nutrient level, the relationship between the RD and temperature (*t*) was fitted by a linear equation in which *a* is the intercept with the *y*-axis and *b* is the slope: RD = *a* + *bt* (Jarošík *et al*. 2004). The LDT, i.e. base temperature, at which the RD ceases (i.e. RD = 0), was then calculated as LDT = −*a*/*b*. The values were calculated separately for individual nutrient levels. The SET thus corresponds to 1/*b*, which indicates the number of DD above the LDT. Thermal window was expressed as the difference between the LDT, and the temperature at which the maximum RD was recorded.

The effect of temperature and nutrients was tested using analysis of variance (ANOVA). A square-root transformation was used to normalize the distributions of plant heights, total biomasses and root/shoot ratios. Logarithmic transformation was similarly used for the data on the time between appearance of the first and seventh pair of stem leaves.

## Results

**Figure 1.**
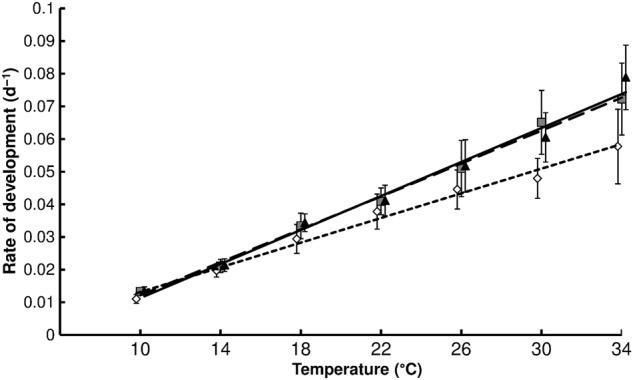
Dependence of the RD of *A. artemisiifolia* seedlings measured as 1/(time from the appearance of the first and seventh pair of stem leaves) on temperature; continuous line and black triangles indicate 100 % Knop solution, dashed line and grey squares 50 % solution, dotted line and white diamonds 10 % solution and bars indicate SD; for the regression equations, coefficient of determination (*R*^2^) and calculated values—LDT and SET, see Table 1.

Both the decrease in temperature and availability of nutrients resulted in a decrease in the RD of the seedlings (Fig. 1) and increase in the time from the appearance of the first and seventh pair of stem leaves (Fig. 1). The slopes of the regression lines arising from the three nutrient levels are significantly different [analysis of co-variance (ANCOVA), *F* = 12.37, *P* = 0.001]. The highest temperature of 34 °C resulted in 5.1, 5.3 and 5.8 times faster development than at 10 °C for the low, moderate and high nutrient levels, respectively. A decrease in the availability of nutrients also resulted in a decrease in the LDT from 5.4 to 3.0 °C (Table 1) and increase in the SET needed to complete this stage in the development of the plants from 392.5 to 546.9 DD (Table 1). Consequently, the thermal window for development was increased from 28.7 °C when the plants were provided with a full nutrient supply to 31.0 °C when provided with the weakest nutrient supply.

**Table 1.** Dependence of the RD on temperature, *t*, fitted using linear regression. The table shows regression equations, *R*^2^, LDT, LDT in °C and SET, SET in DD for nutrient levels equal to 10, 50 and 100 % Knop solution.

Nutrients (% Knop solution)	Regression equation	*R*^2^	LDT (±SD) (°C)	SET (DD)
10	RD = 0.0018*t* − 0.006	0.99	3.01 ± 2.13	546.9
50	RD = 0.0024*t* − 0.012	0.99	4.89 ± 1.29	411.8
100	RD = 0.0025*t* − 0.014	0.98	5.35 ± 2.06	392.5

**Figure 2.**
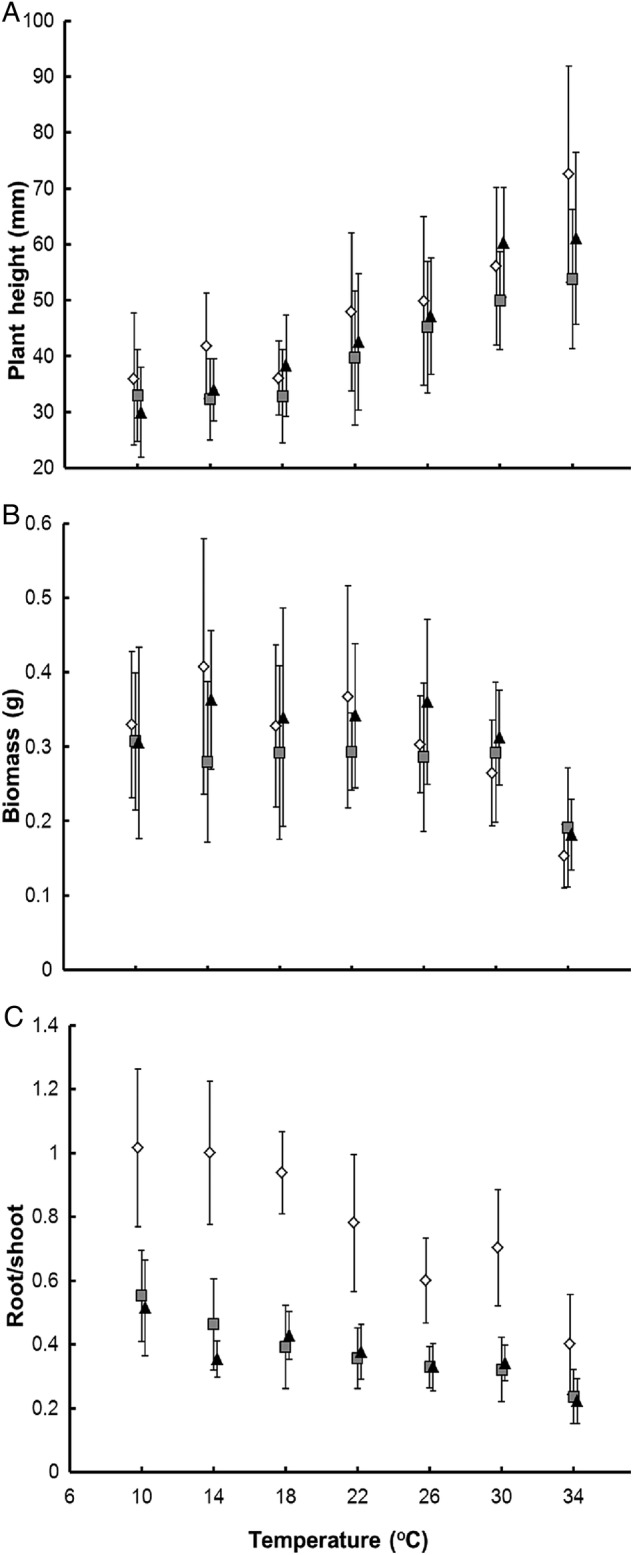
The height (A), biomass (B) and root/shoot ratio (C) of *A. artemisiifolia* seedlings at the seventh pair of stem leaves stage recorded at different temperatures. For significance of these dependences, see Table 2. Black triangles indicate 100 % Knop solution, grey squares 50 % solution, white diamonds 10 % solution and bars indicate SD.

The seedlings grew taller with increase in temperature and decrease in nutrient availability (Table 2, Fig. 2A). Despite their rather quick development and increase in stem height, a marked decrease in biomass was recorded for seedlings grown at the highest temperature of 34 °C (Table 2, Fig. 2B). With decrease in nutrients and decrease in temperature, the seedlings allocated more biomass to roots (Table 2, Fig. 2C).

**Table 2.** Effects of temperature and nutrient level on (i) the RD of *A. artemisiifolia* seedlings, expressed as the time between the appearance of the first and seventh pair of stem leaves, (ii) SET (those above the LDT) between the appearance of the first and seventh pair of stem leaves, (iii) time between the appearance of the first and seventh pair of stem leaves, (iv) height, (v) biomass and (vi) root/shoot ratio of harvested plants. Significance of the effects was tested using ANOVA, significant values are in bold. For all the variables tested, the numbers of degrees of freedom for the effect of temperature, nutrients, the interaction and residuals were equal to 1, 1, 1 and 284, respectively.

	Temperature		Nutrients		Temperature × nutrients	
	*F*	*P*	*F*	*P*	*F*	*P*
RD	2191.33	**<0**.**001**	63.8	**<0**.**001**	36.5	**<0**.**001**
SET (DD)	3.5	0.062	431.3	**<0**.**001**	24.1	**<0**.**001**
Developmental time (days)	932.1	**<0**.**001**	13.9	**<0**.**001**	3.6	0.059
Final seedling height	206.8	**<0**.**001**	4.8	**0**.**029**	0.4	0.555
Total biomass	50.6	**<0**.**001**	0.5	0.468	2.6	0.106
Root/shoot ratio	188.6	**<0**.**001**	265.6	**<0**.**001**	13.3	**<0**.**001**

## Discussion

This experiment revealed a pronounced decrease in developmental rate of *A. artemisiifolia* seedlings when reared at low temperatures and provided with weak solutions of nutrients. This suggests that physiological mechanisms at an early stage in their development contribute to shaping the current distribution of this species in the Czech Republic. The negative response to low temperature is in accordance with its current distribution only in the warmest parts of the country (Jehlík 1998; Pyšek *et al*. 2012*a*). This corresponds to the distribution pattern in Austria (Essl *et al*. 2009), but differs from that in Hungary where the occurrence correlates with low temperatures (Pinke *et al*. 2011, 2013). As the spring and summer temperatures in Hungary are within the thermal window identified by our experiments, this might indicate the importance of other environmental factors such as precipitation and soil characteristics in determining the distribution in this country (Pinke *et al*. 2011). The spring and summer temperatures in most of the Czech Republic are also within the common ragweed's thermal window, but at low temperatures the plants' development is delayed. For *A. artemisiifolia*, an opportunistic species confined to open, disturbed and nutrient-rich ruderal sites and arable land (Pyšek *et al*. 2012*a*), the above factors are likely to influence its already rather poor competitive ability (Leskovšek *et al*. 2012*a*) in regions where temperatures and nutrient supply are suboptimal, and adversely affect the early stage of population establishment.

The maximum rate of leaf development of seedlings of *A. artemisiifolia* was recorded in our experiment at 34 °C, which is close to the 31.7 °C reported by Deen *et al*. (1998*b*) and the maximum rates of development are also similar. Here it needs to be noted that the rate of seedling development increased up to the highest temperature we used, and testing the response to temperatures above 34 °C was not possible due to the technical limitations of the growth chambers. Nevertheless, the abrupt decrease in biomass recorded at the highest temperature and taking into account the results of Deen *et al*. (1998*b*) it is reasonably certain that 34 °C is indeed likely to be the maximum temperature at which *A. artemisiifolia* seedlings can develop. Our results also reveal that the optimum temperature for leaf development is much higher than that for seed germination, which is 18.3 °C (Leiblein-Wild *et al*. 2014). On the other hand, the temperature range of germination, 32.5 °C, i.e. from 2 to 34.5 °C, is similar to that for seedling development (Leiblein-Wild *et al*. 2014).

The width of the thermal window for seedling development varied from 28.7 to 31.0 °C depending on the nutrient supply, which differs from the theoretical prediction based on the biochemical kinetics of metabolism of a constant width of 20 °C (Gillooly *et al*. 2002; Charnov and Gillooly 2003). The deviation from this theoretical prediction, as well as the recorded variation in the RD, LDT and SET, is associated with the availability of nutrients; to the best of our knowledge, this is the first study assessing the effect of an environmental factor on thermal constants of a plant. Still, there are some commonalities if our results are compared with those for insects; the increased rate of seedling development at high nutrient supply is analogous to faster development of ladybirds provided with either more and/or better quality food (Hodek *et al*. 2012; Jarošík *et al*. 2014). For plants, the accelerating effect of nutrients on the development of *A. artemisiifolia* corresponds to the faster leaf development previously recorded at higher levels of radiance for this species (Deen *et al*. 1998*b*), or the increase in the rate of leafing and tillering of the tropical grass *Brachiaria brizantha* recorded when provided with nitrogen (N) and sulfur (S) fertilizers (de Bona and Monteiro 2010). Unlike in aphids and ladybirds (Dixon *et al*. 2013; Jarošík *et al*. 2014), the increased nutrient supply did not result in a decrease in the LDT; on the contrary, nutrient-limited seedlings of the common ragweed had lower LDT. However, it needs to be noted that we derived the LDT from a linear model of plant development and possible non-linearity may occur at temperatures <10 °C. The thermal window of *A. artemisiifolia* is also very wide compared with experimentally obtained windows of a large set of both native and invasive species in central Europe (L. Moravcová and H. Skálová, unpubl. data). Whether a flexible thermal window is a trait associated with invasiveness, which resulted from the evolution of highly invasive genotypes of *A. artemisiifolia* due to seed-mediated gene flow promoted by agricultural disturbance during the westward expansion of human populations in the USA (Martin *et al*. 2014), requires further study. The same holds for possible development of genotypes with wider thermal windows or those shifted towards lower temperatures driven by the existence of locally adapted genotypes, similar to those reported to occur in *A. artemisiifolia* for salinity (DiTommaso 2004).

The faster increase in height of *A. artemisiifolia* seedlings recorded at high temperatures may prevent them from being shaded by neighbouring vegetation, and increase the competitive ability of plants in early stages of population establishment. On the other hand, the tall seedlings that developed at high temperatures weighed less and were weak plants, which may have an opposite effect. In addition, the competitiveness at high temperatures is probably further decreased by reduced allocation to the roots, which constrains nutrient acquisition. The negative effect of nutrient limitation on the performance of *A. artemisiifolia* and the interaction of nutrient availability with temperature explains why this species is confined to nutrient-rich disturbed habitats (Chauvel *et al*. 2006; Essl *et al*. 2009; Šikoparija *et al*. 2009).

## Conclusions

We found that *A. artemisiifolia* plants grow within a temperature range exceeding the 20 °C thermal window, predicted based on the biochemical kinetics of metabolism. The LDT and SET were influenced by growing condition, which contradicts the thermal constant concept. Our results highlight temperature as the main determinant of common ragweed's distribution and identify nutrient availability as a factor that results in the realized niche being smaller than the fundamental niche; both of which need to be taken into account when predicting the future spread of *A. artemisiifolia*.

## Sources of Funding

This research was funded by grants GACR 206/09/0563 and 14-36098G, long-term research development project no. RVO 67985939 from the Czech Academy of Sciences, and by the Praemium Academiae award to P.P.

## Contributions by the Authors

Conceived and designed the experiments: H.S., L.M., P.P. and A.F.G.D. Performed the experiments: H.S. and L.M. Analysed the data: P.K. and H.S. Contributed reagents/materials/analysis tools: H.S., L.M., P.P. and P.K. Wrote the paper: H.S., L.M., P.P., P.K. and A.F.G.D.

## Conflict of Interest Statement

None declared.
