# Corrigendum

**DOI:** 10.1093/aobpla/plv074

**Published:** 2015-07-27

**Citation**: Skálová H, Moravcová L, Dixon AFG, Kindlmann P, Pyšek P. 2015. Effect of temperature and nutrients on the growth and development of seedlings of an invasive plant. *AoB PLANTS* **7**: plv044; doi:10.1093/aobpla/plv044

The authors would like to apologize for the omission of the following acknowledgement:

We acknowledge support from EU COST Action FA1203 ‘Sustainable management of *Ambrosia artemisiifolia*’.

